# Organ System Crosstalk in Cardiometabolic Disease in the Age of Multimorbidity

**DOI:** 10.3389/fcvm.2020.00064

**Published:** 2020-04-28

**Authors:** Yumiko Oishi, Ichiro Manabe

**Affiliations:** ^1^Department of Biochemistry and Molecular Biology, Nippon Medical School, Tokyo, Japan; ^2^Department of Disease Biology and Molecular Medicine, Chiba University Graduate School of Medicine, Chiba, Japan

**Keywords:** organ crosstalk, chronic inflammation, multimorbidity, metabolic syndrome, cardiorenal syndrome, heart failure, somatic mutation, clonal hematopoiesis of indeterminate potential (CHIP)

## Abstract

The close association among cardiovascular, metabolic, and kidney diseases suggests a common pathological basis and significant interaction among these diseases. Metabolic syndrome and cardiorenal syndrome are two examples that exemplify the interlinked development of disease or dysfunction in two or more organs. Recent studies have been sorting out the mechanisms responsible for the crosstalk among the organs comprising the cardiovascular, metabolic, and renal systems, including heart–kidney and adipose–liver signaling, among many others. However, it is also becoming clear that this crosstalk is not limited to just pairs of organs, and in addition to organ–organ crosstalk, there are also organ–system and organ–body interactions. For instance, heart failure broadly impacts various organs and systems, including the kidney, liver, lung, and nervous system. Conversely, systemic dysregulation of metabolism, immunity, and nervous system activity greatly affects heart failure development and prognosis. This is particularly noteworthy, as more and more patients present with two or more coexisting chronic diseases or conditions (multimorbidity) due in part to the aging of society. Advances in treatment also contribute to the increase in multimorbidity, as exemplified by cardiovascular disease in cancer survivors. To understand the mechanisms underlying the increasing burden of multimorbidity, it is vital to elucidate the multilevel crosstalk and communication within the body at the levels of organ systems, tissues, and cells. In this article, we focus on chronic inflammation as a key common pathological basis of cardiovascular and metabolic diseases, and discuss emerging mechanisms that drive chronic inflammation in the context of multimorbidity.

## Introduction

It is now well understood that organ systems are tightly interlinked and coordinately respond to internal and external demands and disturbances, thereby dynamically maintaining the body's homeostasis. For instance, the cardiovascular system redistributes the blood supply to tissues that demand more oxygen (e.g., skeletal muscle during exercise or the gut after a meal) via regulatory mechanisms that are local and intrinsic to the system as well as in response to signals from the nervous and endocrine systems (1). While such close interactions among systems are crucial for protecting organs and promoting health [e.g., exercise-induced cardioprotection, presumably through crosstalk between the heart and skeletal muscle (2)], they may also promote and expand pathologies to multiple organs. Indeed, a number of clinical studies have shown close associations among the progression of cardiovascular, metabolic, and kidney diseases, which is indicative of the mechanistic links between those diseases. Metabolic syndrome and cardiorenal syndrome are coined terms that highlight the strong clinical associations among disorders of the cardiovascular and metabolic systems. Metabolic syndrome is characterized by central obesity plus hypertension, insulin resistance, and dyslipidemia. The components of metabolic syndrome themselves are known to strongly associate with each other in obese subjects (3, 4), and they synergistically increase the risks for a variety of diseases, including coronary artery disease, diabetes, heart failure, and some cancers. Cardiorenal syndrome reflects conditions in which failure of either the heart or kidney leads to, or accelerates, failure of the other organ (5). In addition, other syndromes have been proposed to account for the expanding clinical and experimental evidence of complex communication among organs and organ systems, including cardiohepatic, hepatorenal, and intestinal–renal syndromes, to name a few examples.

One key reason for the recent growth in the interest in organ and organ system crosstalk is that there are increasing numbers of patients with multimorbidity, which has a great impact on disease management and health care costs (6). Multimorbidity is defined as the coexistence of two or more chronic conditions, which must be a non-communicable disease (NCD) of long duration, such as cardiovascular disease (CVD); cancer; a mental health condition of long duration, such as a mood disorder or dementia; or an infectious disease of long duration (7, 8). Nearly three in four individuals aged 65 years and older have multimorbidity, highlighting the need to direct effort toward the development of clinical strategies and practices and a health care system suited for this rapidly growing clinical burden (9). For that, it is undoubtedly necessary to elucidate the mechanisms that connect individual diseases and promote multimorbidity as a unified condition, not as the simple coexistence of individual diseases. It is particularly important to develop novel therapeutic strategies to treat chronic diseases in patients with multimorbidity, which are in fact the majority of current patients.

A key driver of the increase in multimorbidity is aging (6, 10), though obesity and a lifestyle with diminished physical activity also contribute to the risk of multimorbidity (10). We can therefore anticipate that with the continued graying of society, the significance of multimorbidity will increase. Many chronic diseases, including cardiovascular, metabolic, renal, and pulmonary diseases, share the same risk factors. However, the coexistence of multiple diseases is not simply the result of several diseases being separately and independently induced by their common risk factors. Instead, the development of any one of the diseases greatly impacts the development and course of the coexisting diseases (5). In other words, multimorbidity develops upon a nexus of multiple organ and systemic dysfunctions.

**Figure 1:**
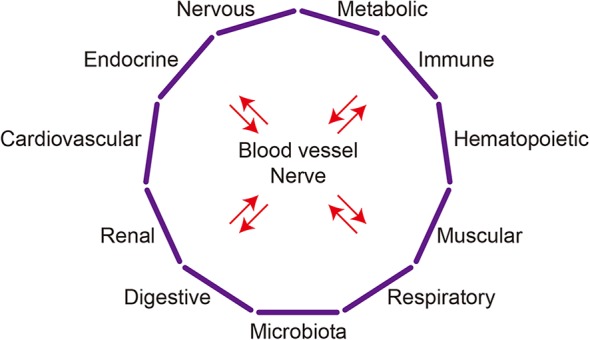
Organ system crosstalk. The major organ systems discussed in this article are depicted schematically. Complex interactions among organs systems underlie the crosstalk between any two organs.

The communication between organs is mainly mediated by two types of wiring: blood vessels and nerves (Figure 1). Nerves can directly establish connections between two or more organs, while signaling factors transferred through the blood circulation can selectively affect particular organs expressing specific receptors or as a result of the organ's proximity to the blood vessel connection.

Although studies have mainly focused on the reciprocal interactions between pairs of organs and the diseases affecting them, the crosstalk between two organs may involve mechanisms that go beyond those two organs. For instance, the communication between the heart and kidneys in cardiorenal syndrome may involve the brain, adrenal glands, and bone marrow (11). It is likely that any crosstalk between two organs involves additional organs and, indeed, whole-body responses mediated by organ systems. Accordingly, organ crosstalk is established not only between two organs but among multiple organ systems, such as the cardiovascular, nervous, endocrine, metabolic, and immune systems. In that sense, “organ system crosstalk” may better illustrate the complex interactions among organs (Figure 1). Moreover, the mechanisms that realize organ crosstalk function at the system, tissue, cell, and molecular levels.

In this article, we will briefly overview our current understanding of the mechanisms involved in organ–organ and organ–system crosstalk, using metabolic syndrome and heart failure as examples. We will then discuss the mechanisms that enable organ system crosstalk, focusing in particular on chronic inflammation as the convergence point for intersystem crosstalk. Finally, we will discuss the future directions of the research on organ (system) crosstalk, the aim of which is a better understanding of the processes and the development of novel therapeutic and diagnostic strategies.

## Metabolic Syndrome and Organ Crosstalk

### Adipose Tissue Is a Key Regulator of Systemic Metabolism

Metabolic syndrome is characterized as a combination of risk factors that culminate in adverse outcomes from cardiovascular and metabolic diseases, including type 2 diabetes and CVD. The major components of metabolic syndrome are central obesity, hypertension, insulin resistance, and dyslipidemia (12). The clustering of the components of metabolic syndrome not only increases the risk for cardiovascular and metabolic diseases but also points to a mechanistic link among these components. Central or abdominal obesity is considered to be crucial to the clustered development of the related dysfunctions. As a result of the discovery of various mediators produced by adipose tissue, collectively called adipokines or adipocytokines (e.g., leptin and adiponectin), adipose tissue is considered to be an active endocrine organ. The altered production of adipokines by obese visceral adipose tissue is thought to be a key mechanism that promotes insulin resistance, hypertension, and dyslipidemia as well as CVD. Accordingly, crosstalk between visceral adipose tissue and distant organs and tissues, including the heart, liver, pancreas, and blood vessels, is central to the concept of metabolic syndrome.

The two major types of adipose tissue are white adipose tissue (WAT) and brown adipose tissue (BAT). The major fat depots contain WAT, which is a reservoir of energy stored as triglycerides. BAT, on the other hand, dissipates energy for heat production. To properly store and deliver energy, WAT responds to a variety of metabolic signals, including circulating nutrients and metabolites as well as neural input, cytokines, and other mediators from distant organs. In addition, to control the storage and supply of energy, WAT also actively controls systemic energy homeostasis by regulating energy expenditure, satiety, and release of glucoregulatory hormones (e.g., insulin and glucagon) from the pancreatic islets (13). While the production and secretion of adipokines are one mechanism by which organ crosstalk is mediated, other metabolic and neural mechanisms are also crucially involved. It is likely that multiple communication pathways have evolved to enable adipose tissue to control systemic metabolic homeostasis by dynamically responding to the body's metabolic demands and nutrient flux, functioning as the center of a network controlling systemic metabolism. Adipose tissue thus acts as a central regulator of energy homeostasis by acting as an active endocrine and energy-supply organ. However, these same pathways may also enable pathological communication. We will overview some of those pathways in the following sections. While we are focusing on visceral adipose tissue, which is WAT, recent studies have suggested that BAT and beige cells (brown adipocyte-like cells within WAT) are also actively involved in organ crosstalk.

### Immunometabolic Crosstalk in Metabolic Syndrome

**Figure 2:**
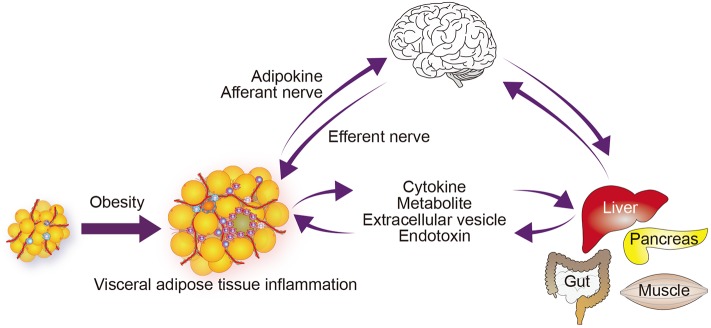
Immunometabolic organ crosstalk in obesity. Obesity induces chronic inflammation in visceral adipose tissue (17, 18). This inflammation increases the release of free fatty acids and some adipokines, which modulate the function of metabolic and endocrine organs and enhance inflammatory processes in those organs (19). Signals from the affected organs in turn affect metabolic and inflammatory signals and processes within the adipose tissue. Obesity may also directly activate or enhance inflammation. The central and autonomic nervous systems receive information from peripheral metabolic organs, and their output affects metabolism and inflammation in those organs (20–23). Obesity also induces inflammation in some regions of the CNS, including the hypothalamus, which may alter its function (24). Although not depicted in the figure, the gut microbiome contributes to the organ network by supplying endotoxin and a variety of metabolites.

WAT, particularly visceral adipose tissue, contains a large number of immune cells. These immune cells appear to be integral to adipose tissue physiology, contributing to the clearance of cellular debris, the buffering of lipids, and the expansion of the fat depot (14–16). However, a hallmark of obese visceral adipose tissue is inflammation within the tissue, and immune cells play active and crucial roles in those immune responses (17, 18). Local immune cells are able to modulate the activities of adipocytes and other cells within adipose tissue, thereby altering the tissue's function. Moreover, proinflammatory cytokines produced by these immune cells may contribute to inflammation in distant tissues (Figure 2). Adipose tissue immune cells may also respond to circulating molecules, including gut-derived danger signals, such as lipopolysaccharides (LPS), as well as to neuronal signals (25, 26). Adipose tissue immune cells can thus act as sensors of local, systemic, and more distant signals (27).

In addition to adipokines, metabolites, such as free fatty acids and lipid mediators released from adipose tissue all affect systemic insulin resistance and inflammation in distant organs (19). Free fatty acids are generated from triglyceride within adipocytes (the process is called lipolysis) and transferred to distant tissues through the circulation. Obesity augments lipolysis, in part by impairing insulin signaling, which increases circulating free fatty acid levels. Within the pancreatic islets, β cells sense the excess free fatty acids and initiate an inflammatory cascade by recruiting circulating monocytes. These monocytes differentiate into proinflammatory macrophages, leading to islet inflammation, which, in turn, causes β cell dysfunction (18, 28, 29). Accordingly, adipose tissue inflammation is linked to inflammation within pancreatic islets in part through metabolites (e.g., free fatty acids) (Figure 2). In addition, proinflammatory molecules such as tumor necrosis factor (TNF)-α and galectin-3, which are produced by adipose tissue and induce insulin resistance, and interleukin (IL)-1β, which impairs β cell function, may also cause metabolic dysfunction in distant tissues (30–32). These findings highlight the complex interactions between metabolism and immunity (immunometabolism) in systemic metabolic homeostasis and dysfunction.

### Neural Control of Metabolism and Inflammation

The nervous system is another key coordinator of adipose tissue and other metabolic tissues in systemic metabolism. For instance, neural signals from WAT modulate hypothalamic leptin sensitivity (20, 21). Conversely, leptin signaling activates lipolysis via sympathetic nerves that innervate the WAT (Figure 2). Neural mechanisms also link the liver and adipose tissue (22). Elevation in circulating amino acids activates mTOR signaling in the liver, leading to hypertriglyceridemia, mainly due to downregulation of lipoprotein lipase expression in adipose tissue. This amino acid–lipid metabolism link is mediated by a neuronal pathway consisting of the afferent vagal nerve from the liver and efferent sympathetic nerves to adipose tissue.

Immune cells, particularly macrophages, interact with nerves and mediate or modulate some neural effects on metabolic tissues. In the liver, for example, the vagus nerve constitutively suppresses IL-6 expression in Kupffer cells by activating α7-nicotinic acetylcholine receptors (23). Central insulin signaling inhibits vagus nerve activity, promoting IL-6 production in Kupffer cells, which in turn suppresses gluconeogenic genes in hepatocytes. Overall, Kupffer cells mediate the vagal response to central insulin action with hepatic glucose production. After a liver injury caused by partial hepatectomy, hepatocyte proliferation is stimulated by the vagus nerve through upregulation of IL-6 production in liver macrophages (33). In these models, macrophages are an essential intermediary supporting vagal nerve actions in the liver. In addition, vagal nerve activation induced by *Pten* deletion in insulin receptor-expressing neurons promotes M2-like activation in macrophages in peripheral tissues, including adipose tissue, which results in greater insulin sensitivity in high fat-diet (HFD)-induced obese mice (34).

Conversely, macrophages can also suppress nerve actions. In WAT, a specialized population of macrophages, termed sympathetic neuron-associated macrophages, wrap around sympathetic nerves (35). These macrophages express a noradrenaline transporter, solute carrier family 6 member 2 (SLC6A2), and a norepinephrine degradative enzyme, monoamine oxidase A (MAOA), and likely to take up and degrade excess noradrenaline. Adipose tissue macrophages in aged mice express higher levels of *Maoa*, reflecting at least in part NLRP3 inflammasome activation (36). It has been suggested that those macrophages inhibit lipolysis activated by sympathetic nerves. This observation also suggests that macrophages sense inflammatory signals and modulate sympathetic nerve activity.

Obesity also induces inflammation in the central nervous system (CNS). Even when administered for only a short period (1–3 days), a HFD induces hypothalamic inflammation in mice and rats (24). Longer periods on a HFD activates inflammation in other regions of the CNS, including the hippocampus, which may reduce leptin sensitivity (37, 38). Inflammation in the CNS thus appears to be an important link between the metabolic and nervous systems. Beyond the metabolic abnormalities, obesity-induced neuroinflammation has also been shown to associate with cognitive dysfunction, anxiety, and depression in mice.

## Heart Failure (HF) is a Systemic Syndrome

### HF and Organ Crosstalk

HF involves several pathological alterations within the heart, exemplified by left ventricular remodeling. However, its development is greatly affected by extracardiac diseases and conditions. Conversely, HF can contribute to the development of dysfunction in other organs. In that sense, HF should be considered a systemic disease. For instance, metabolic syndrome and obesity are risk factors for HF (39). Renal dysfunction also strongly impacts outcome in patients with HF (40, 41). The existence of kidney disease increases the risk of cardiovascular mortality among HF patients by 10-fold, as compared to the general population. More than 40% of patients with chronic HF also have chronic kidney disease (CKD) (42–44). This close association between HF and CKD led to the concept of “cardiorenal syndrome,” which encompasses concomitant bidirectional dysfunction of the heart and kidneys, whereby dysfunction in one organ induces or accelerates dysfunction in the other (45, 46). Recent studies have begun to shed a light on the mechanisms responsible for the organ crosstalk in HF and other heart diseases. What follows is an overview of some of those mechanisms.

### Connective Mechanisms in Cardiorenal Syndrome

A variety of mechanisms that connect heart and kidney disease have been identified, including hemodynamic factors, the renin–angiotensin–aldosterone system (RAAS), sympathetic nervous system, and inflammation (11, 46). For instance, venous congestion with increased renal vein pressure is now considered to be a primary hemodynamic factor that worsens renal function in HF (46, 47). Activation of the RAAS is a shared feature of HF and CKD that promotes both pathologies (46). Overactivation of the sympathetic nervous system is another feature common to both HF and CKD. Inflammation is also known to play important roles in the development of both pathologies and may connect them via circulating cytokines and other proinflammatory molecules, such as endotoxin (48, 49). These connecting mechanisms also interact with each other. RAAS activation increases sympathetic nervous system activity and stimulates inflammation, while inflammation may potentiate the RAAS and sympathetic nervous system (50).

**Figure 3:**
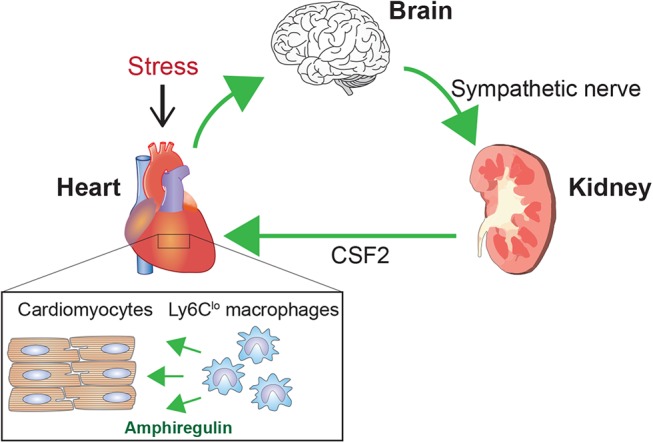
Brain-heart–kidney network in cardiac adaptation. Pressure overload on the left ventricle triggers a cardioprotective mechanism involving the brain, heart, and kidneys (51). Pressure overload activates sympathetic nerves to the kidneys. Within the kidneys, sympathetic nerves stimulate collecting duct epithelial cells, which activate cellular interactions leading to renal production of CSF2/GM-CSF. CSF2 is transferred to the heart via the circulation and activates cardiac tissue-resident Ly6C^lo^ macrophages. These activated Ly6C^lo^ macrophages then play a pivotal role in the adaptive response to the pressure overload. A key cardioprotective mediator produced by Ly6C^lo^ macrophages is amphiregulin (AREG).

New connecting mechanisms are also emerging. For instance, we identified a heart–brain–kidney network that controls the adaptive response to pressure overload in the hearts of mice (Figure 3) (51). Pressure overload in the heart activates renal collecting duct epithelial cells via sympathetic nerves. Within the kidneys, activated communication between collecting duct epithelial cells, tissue macrophages, and endothelial cells leads to secretion of colony stimulating factor 2 (CSF2), which in turn stimulates cardiac-resident macrophages. These macrophages play an essential cardioprotective role in part through production of amphiregulin, an epidermal growth factor ligand. In this scenario, the cardiovascular, nervous, and immune systems cooperate to maintain homeostasis. Such tight cooperative links among these systems may also accelerate dysfunction and pathology within these systems.

### Crosstalk Between CVD and the Bone Marrow

Following myocardial infarction (MI), a large population of monocytes is recruited to the heart and contributes crucially to the processes of inflammation, its resolution, and healing (52). Monocytes are recruited from bone marrow and extramedullary reservoirs, including the spleen (53), and at least a part of these monocytes differentiate into macrophages (54). Dutta et al. reported that MI promotes sympathetic nerve-activated migration of hematopoietic stem and progenitor cells (HSPCs) from the bone marrow to the spleen (55). The migrated HSPCs undergo prolonged amplified extramedullary monocyotopoiesis, which results in enhanced inflammation within atherosclerotic plaque in *Apoe*^−/−^ mice.

Sympathetic signaling is a crucial regulator of HSPC mobilization from the bone marrow to the circulation in various physiological and pathological settings (56). For example, immune cell activation contributes crucially to the development of various models of hypertension, and sympathetic nerve activity appears to play an important role in that activation (57). A central infusion of angiotensin II in rats increases bone marrow sympathetic nerve activity, which parallels increases in blood pressure and systemic inflammation (58). It also activates microglia in the hypothalamus. These findings demonstrate the complex interactions among the hematopoietic, immune, and nervous systems during the development of cardiovascular diseases. However, these interactions appear to converge on inflammation within and beyond the cardiovascular tissues, leading, for example, to neuroinflammation.

## Chronic Inflammation as a Nexus of Multiorgan Disease Progression

As overviewed in the previous sections, organs communicate via several mechanisms, though direct communications are established through two major pathways: the circulation and nervous system (Figure 1). In the blood, diverse molecules, including hormones, cytokines, and nutrients; various types of vesicles containing a variety of molecules; and cells, including immune cells; mediate communication. In addition, the physical and chemical properties of circulation itself, including blood flow, pressure, temperature, and pH, may also convey information. Nerves may connect specific regions or cells in one organ to those in another organ, or with specific nuclei in the brain.

Although direct communications are important, indirect and/or systemic effects also play a role in maintenance of tissue and systemic homeostasis, as well as the spread of dysfunction to multiple organs. As we discussed above, for example, the development of CVD in the context of metabolic syndrome may involve changes in the metabolic, endocrine, nervous, hematopoietic, and renal systems, which in turn affect various organs outside of the cardiovascular system. One common biological process that is activated and modulated by those changes in the multiple systems is inflammation, particularly during disease development.

Chronic inflammation is a common feature of NCDs, including CVD, metabolic disease, and cancer (59). It appears that a key nexus within the complex network of biological processes operating in NCDs is inflammation. As we discussed, chronic inflammation is involved in multiple aspects of organ crosstalk. In NCDs, chronic inflammation may not be confined to the primarily affected organ or tissue. It is more likely to manifest as a multiorgan or systemic process. For instance, inflammation initiated in obese visceral adipose tissue may activate and/or accelerate inflammation in distant tissues, such as liver, pancreas, and arteries. Other common pathological mechanisms are also emerging, including altered metabolism and somatic mutations. Notably, these mechanisms are also tightly linked to inflammation. In the following sections, we will discuss emerging biological mechanisms that closely interact with inflammation.

While chronic inflammation has been mainly studied in the context of disease progression, in many conditions, it is likely initiated as an adaptive response to stress, just as acute inflammation is. During an acute infection, insulin resistance is thought to be induced to mobilize nutrients to fuel immune system activation, which is energy demanding (60, 61). It is possible that the inflammatory program has also evolved to cope with stress unrelated to infection or tissue injury, such as metabolic and physical stress. For instance, obesity activates inflammation within adipose tissue, resulting in insulin resistance. It has been proposed that this induction of insulin resistance is initially adaptive and promotes nutrient mobilization from adipose tissue to reduce adiposity (25). Inflammatory signals and immune cells have also been shown to play physiological roles within adipose tissue. For instance, proinflammatory signaling in adipocytes is essential for healthy expansion of adipose tissue (14). Macrophages are important for local lipid metabolism (62). These suggest that inflammatory processes and cells have essential homeostatic and adaptive functions within adipose tissue. However, unless the inflammatory process is properly controlled and resolved, it eventually becomes maladaptive. In the case of obesity, the initial activation of inflammation within adipose tissue promotes systemic metabolic abnormalities and activation of inflammation in various organs. That said, the emerging tight and intricate connections between the regulatory mechanisms governing inflammation and many other systems, including the metabolic, endocrine, and nervous systems, suggest that these connections are integral to the proper physiological and adaptive functions of the tissues as well as the body as a whole. We therefore need to further elucidate the physiological and homeostatic functions of chronic inflammation to better map the organ system interactions and their biological functions.

## Emerging Mechanisms that Converge on Chronic Inflammation

### Immunometabolic Connections

The field of immunometabolism has mainly focused on two facets of the complex crosstalk between immunity and metabolism: the immune contribution to metabolic disease and the metabolic regulation of immune cell function. Regarding the former, studies have shown that immune cells play important roles in both the physiology and pathology of major metabolic tissues, including adipose tissue, liver, skeletal muscle, and pancreatic islets (30). A key pathological mechanism is chronic inflammation. Systemic metabolic abnormalities, such as obesity and diabetes, initiate, potentiate, and sustain chronic inflammation in those tissues. As we discussed earlier, even in a lean healthy state, visceral adipose tissue is particularly rich in immune cells, which appear to be integral to the physiological function of the tissue. In obesity, however, immune cells, including many newly accumulated proinflammatory cells, lead active inflammatory processes and promote adipose tissue dysfunction. Importantly, this inflammation is not quarantined within the adipose tissue and influences signaling and metabolic and inflammatory processes in distant tissues in the body. This appears to be a key pathological mechanism underlying the propagation of organ dysfunction in obesity (Figure 2).

The other facet of the field of immunometabolism is the metabolic regulation of immune cells. Changes in cellular metabolism are important for differentiation, activation, suppression, survival, and death of immune cells (63). Metabolic reprogramming appears to be tightly connected to immune cell signaling and epigenetic regulatory mechanisms. Moreover, it is unlikely that the crosstalk between metabolism and immune signaling is confined within these cells. Instead, it appears to be open to the microenvironment surrounding the cells. This metabolic microenvironment may fuel or modulate such metabolic regulation of immune cell activity and fate. Nutrients and metabolites, including those produced by gut microbiota, affect immune cell activation (64, 65). For instance, uptake of lipids for mitochondrial fatty acid oxidation (FAO) is essential for survival of CD8^+^ tissue-resident memory T cells (66). Transition of macrophages from the pro-inflammatory to pro-resolution phenotype depends on activation of unsaturated fatty acid synthesis, and exogenous polyunsaturated fatty acids can promote this transition (67). Such potential crosstalk among the metabolic microenvironment and immune cell metabolism and the cells' activation may comprise additional organ–organ or organ–whole body linkages (60, 68).

Obesity and insulin resistance may modulate immune responses by altering the metabolic microenvironment, for example, by altering the availability of nutrients such as lipids, including phospholipids within cell membranes, and other tissue metabolites. Mauro et al. recently reported that obesity promotes differentiation of effector memory T cells that traffic into nonlymphoid and inflamed tissue (69, 70). Mechanistically, saturated fatty acids such as palmitate activate Akt signaling and FAO, which supports effector memory T cell differentiation and illustrates the link between the metabolic microenvironment and immune cell activation. However, this potentially important organ/system crosstalk pathway via the metabolic environment has not been well studied. It also remains unclear to what extent changes in the systemic and tissue metabolic environment modulate immune cell function by altering their cellular metabolism, as metabolites and metabolic signals may directly affect immune signaling pathways independently of changes in cellular metabolism.

Other mechanistic links between immunity and metabolism have also been identified. For instance, some cytokines can control both immunity and metabolism. A prime example is leptin. This protein is produced by adipose tissue and controls appetite and other central regulators of energy homeostasis (71). However, leptin also exhibits proinflammatory actions in part by acting on various immune cells, including macrophages, natural killer (NK) cells, and lymphocytes (72). Conversely, inflammatory cytokines have also been shown to control metabolism. IL-6, for instance, controls lipid metabolism (73). IL-6 is also secreted from skeletal muscle during exercise and controls systemic metabolism (74). Other cytokines, including IL-1β (75, 76), IL-33 (77), and adiponectin (78), also exhibit bifunctionality in immunity and metabolism. This suggests that these cytokines may act as coordinators of immunity and metabolism.

Intracellular signaling pathways activated by cytokine receptors and metabolic sensors form an additional complex layer of crosstalk. A variety of receptors, including cytokine receptors, T cell receptor, and pattern-recognition receptors, such as TLRs, are linked to Akt and mTOR, which are central signaling hubs for cellular metabolism and also receive information from receptors for metabolic hormones, such as insulin (79, 80). Regulation of the Akt and mTOR pathways by cytokine receptors and immune sensors is integral to the metabolic reprograming that supports immune cell activation. Conversely, metabolic hormone receptor signaling may also affect immune cell function. For instance, leptin's activation of its receptor activates the PI3K-Akt-mTOR pathway, which increases the glycolysis that supports proinflammatory cytokine production (81).

**Figure 4:**
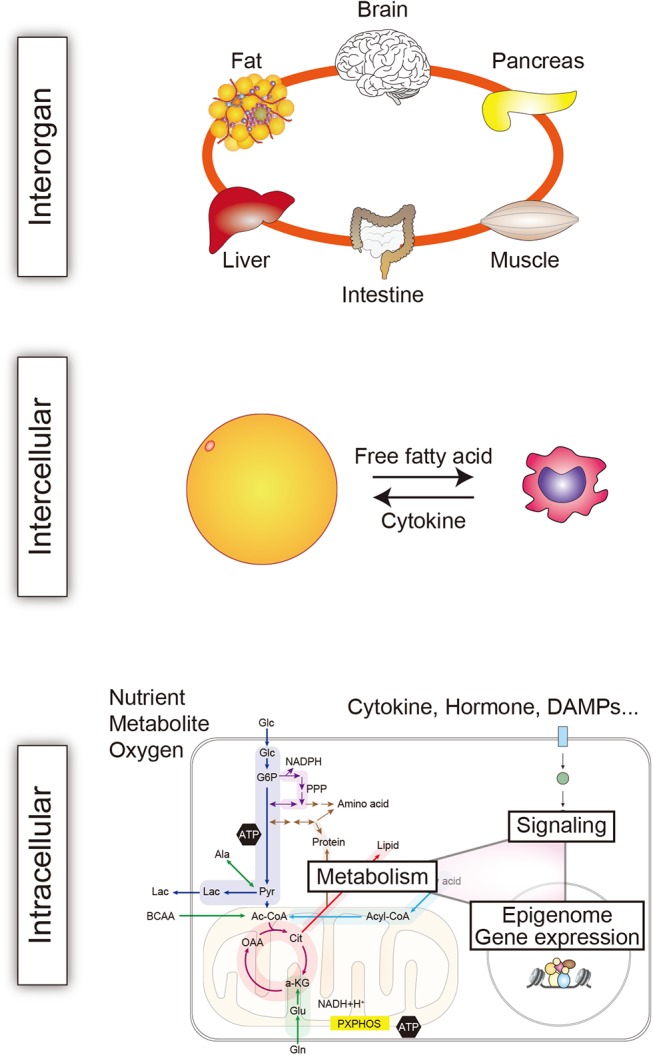
Immunometabolic crosstalk at multiple levels. The crosstalk between immune and metabolic mechanisms operates at multiple levels in the body to maintain homeostasis. The intricate crosstalk also crucially contributes to the development of systemic, tissue, and cellular dysfunction and disease. One key pathological mechanism mediated by immunometabolic crosstalk is chronic inflammation. The interaction between immune and metabolic cells is exemplified here by interaction between a macrophage and an adipocyte (82).

The connections between the mechanisms underlying immunity and metabolism are multifold and operating at the multiple levels, extending from the molecular level to the body as a whole (Figure 4). Such extensive crosstalk appears to be crucial for homeostasis and adaptation to stress. However, this crosstalk may also activate, advance, and sustain chronic inflammation. In addition, recent studies have established that metabolites generated by gut microbiota can modulate immune responses (83). This adds another layer of linkage in immunometabolism. Collectively, the findings summarized above make it clear that the immune and metabolic systems are highly intertwined, and these extensive connections appear to be crucial for maintenance of homeostasis. We anticipate that future studies will unravel the pathological functions of the immunometabolic connections and identify novel therapeutic targets.

### Neuroimmune Communication in Chronic Inflammation

An important connection between the nervous system and pathological processes in NCDs is neuroimmune communication. Recent studies have unraveled the tight integration of the reciprocal communication between the immune and nervous systems: neural signaling is activated by inflammation and immune cell function is affected by neurotransmitters (84).

Vagal afferent nerves express pattern-recognition receptors, such as TLRs, and respond to inflammatory molecules, such as pathogen-associated molecular patterns (PAMPs) and damage/danger-associated molecular patterns (DAMPs) (84–86). They also express cytokine receptors and respond to inflammatory signals from immune and other peripheral cells. Conversely, immune cells express receptors for neurotransmitters, including acetylcholine and noradrenaline, which respond to neural input; moreover, immune cells, such as T cells, can produce neurotransmitters. For instance, a population of T cells respond to the vagus nerve and produce acetylcholine, which in turn inhibits acetylcholine receptor-expressing macrophages, thereby relaying neural signals (87). Acetylcholine is also produced by B cells and NK cells and controls immune cell recruitment (88, 89). Noradrenaline, dopamine, GABA, and VIP are also produced by immune cells (84).

**Figure 5:**
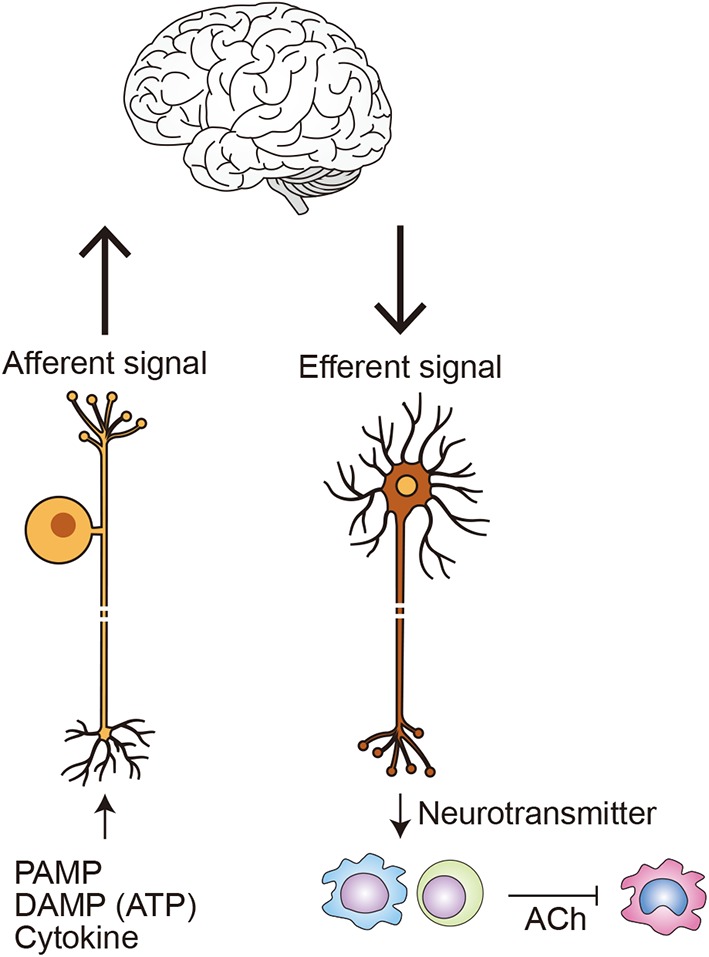
Neuroimmune communication in inflammation. Afferent neurons monitor peripheral immune status by sensing PAMPs, such as LPS; DAMPs, such as ATP; and cytokines, such as TNF-α and IL-1β (84). Efferent nerves release neurotransmitters, such as noradrenaline and acetylcholine (ACh), which directly or indirectly activate a variety of immune cells, including macrophages, dendritic cells, and lymphocytes. Within the best-studied circuit of neuroimmune communication, the cholinergic anti-inflammatory pathway, inflammation in the periphery is detected by afferent vagal neurons. The information is processed in the brainstem, initiating the efferent arm of anti-inflammatory pathway. Within the spleen, noradrenaline released from efferent nerves activates β2-adrenergic receptors on choline acetyltransferase-expressing T cells. The affected T cells in turn release ACh, which suppresses TNF-α production in nicotinic α7-nicotinic receptor-expressing macrophages, in part by inhibiting NF-κB signaling (84, 86). Other stimuli that also activate the anti-inflammatory pathway includes PI3 kinase activation within inulin-expressing neurons (34).

A growing number of pathways that tightly connect the immune and nervous system have been identified. One of the best-studied pathways is a cholinergic anti-inflammatory reflex pathway (Figure 5) (86, 90). In this pathway, inflammation is detected by vagal afferent neurons in peripheral tissues. That information is transmitted to the solitary nucleus in the brainstem, where it is processed, resulting in vagal efferent (parasympathetic) outflow to the sympathetic ganglia that innervate the spleen and release noradrenaline there (84). Noradrenaline activates β2-adrenergic receptors on choline acetyltransferase-expressing T cells, which in turn release acetylcholine. The released acetylcholine suppresses proinflammatory activation of nearby nicotinic α7-receptor-expressing macrophages. The net effect of this anti-inflammatory reflex is inhibition of cytokine release by macrophages in the spleen, which accounts for 90% of the TNF and IL-1 produced in acute endotoxemia (86).

Other reflex pathways that connect the immune and nervous systems have been identified and shown to be important for regulating inflammation in a variety of disease models (91). In animal models, moreover, electrical vagus nerve stimulation appears to be effective against arthritis, irritable bowel disease, and ischemia reperfusion injury in brain, heart, and kidneys, and clinical trials of its use to treat various diseases are underway (84, 92). However, the efficacy of vagus nerve stimulation has so far been evaluated mainly in models of acute inflammation; less is known about its efficacy in chronic disease models, including cardiovascular and metabolic diseases. Wang et al. reported that vagal nerve activation promotes M2-like activation of macrophages in peripheral tissues, ameliorating insulin resistance in obese mice (34). This suggests dysfunction within anti-inflammatory reflex pathways may promote metabolic disease. As discussed, macrophages mediate the effects of the autonomic nervous system on metabolic tissues, while sympathetic nerve activity regulates immune cell activation and mobilization through organ crosstalk during both adaptive and maladaptive responses to cardiac stress. What's more, sympathetic nerve hyperactivity within the spleen may underlie chronic inflammation (93). From these findings, it appears likely that neuroimmune communication is an important regulatory hub mediating the systemic response to stress, and its dysregulation may underlie chronic inflammation in NCDs, though further studies addressing these ideas are definitely needed.

### Somatic Mutations in Blood Cells and CVD

Clonal hematopoiesis is an expansion of blood cells derived from a single hematopoietic stem cell (HSC). Clonal hematopoiesis with somatic mutations was found in 10% of subjects over 65 years of age (94). Notably, clonal hematopoiesis apparently increases with age, as it was observed in only 1% of subjects <50 years old (94–96). A somatic mutation that grants a selective advantage to a particular HSC allows its expansion relative to other HSCs (97), which is thought to lead to clonal hematopoiesis.

Individuals with clonal hematopoiesis of indeterminate potential (CHIP) carry a hematologic malignancy-associated somatic mutation in their blood or bone marrow, but without a hematologic malignancy (98). However, these individuals have an approximately 10-fold greater risk for hematologic cancer than the general population, suggesting that CHIP may represent a premalignant state (94). CHIP is also associated with increased all-cause mortality, though the cause of this higher mortality is not a higher rate of hematological malignancies, but an increased rate for coronary heart disease and ischemic stroke (95, 97). These findings indicate that CHIP is a new and important CVD risk factor.

The association between CHIP and CVD may reflect aging and/or the presence of common risk factors for somatic mutations and CVD. However, it is also possible that CHIP causally contributes to CVD. Recent animal studies support the latter possibility. A cancer driver gene is a gene whose mutation increases net cell growth under the specific microenvironmental conditions within which the cell exists *in vivo* (99). In other words, a driver gene mutation gives a selective advantage to a clone by increasing its survival and/or proliferation. In *Ldlr*^−/−^ mice, transplantation of a mixture of 90% wild-type bone marrow cells and 10% bone marrow cells deficient for the cancer driver gene *Tet2* led to clonal expansion of *Tet2*^−/−^ cells in the blood and accelerated atherosclerotic plaque formation (100). Similarly, C57BL/6J mice carrying the same bone marrow chimera exhibited worsening cardiac remodeling and dysfunction after MI or transverse aortic constriction (101). *Tet2*-deficient macrophages showed enhanced inflammatory responses and expression of proinflammatory cytokines, including IL-1β (100, 102). Hematopoietic deletion of another driver gene, *Dnmt3a*, also enhanced angiotensin II-induced cardiac remodeling and cardiac expression of inflammatory cytokines (102). A mutation in *Jak2* (*Jak2*^V617F^) led to an increased propensity for neutrophil extracellular trap (NET) formation and venous thrombosis (103). Hematopoietic *Jak2*^V617F^ also enhanced atherogenesis in *Ldlr*^−/−^ mice (104) by promoting neutrophil infiltration into early lesions and increasing the necrotic cores and defective efferocytosis (clearance of dead leukocytes) in advanced lesions. These findings suggest that modulation of immune cell function that promotes inflammation is one possible mechanism for CHIP-induced acceleration of cardiovascular pathology (105).

Although studies have so far focused largely on the effects of CHIP on the cardiovascular system, CHIP would be expected to alter inflammatory responses in many tissues and systematically, which would also modulate organ crosstalk. Moreover, in various tissues, immune cells, such as macrophages, have homeostatic and physiological functions that may not directly relate to inflammation. For instance, macrophages are important for maintaining electrical conduction in the heart (106). CHIP might also modulate such functions and alter cardiac tissue homeostasis, though this has not yet been tested.

Somatic mutations are also found in other normal cells. For instance, sun-exposed skin cells carry thousands of point mutations, and in individuals ranging from 55 to 73 years of age, ~25% of those cells carried at least one driver mutation (107, 108). Accordingly, aged sun-exposed skin is a patchwork of evolving clones in which over 25% carry cancer-causing driver mutations while the cells maintain the physiological functions of skin. Similar age-related expansion of clones with driver mutations is observed in other normal tissues, including esophageal epithelium (109). Yizhak et al. recently screened RNA sequencing data from 29 normal tissues for somatic mutations. While sun-exposed skin, esophagus, and lung had a higher mutation burden than other tissues, clonal expansion was detected in all the tissues tested, including the heart (110). This suggests that somatic mutations may contribute to age-associated diseases, though this possibility remains largely unexplored.

### Aging and Organ System Crosstalk

Aging is a key driver of multimorbidity. Aging-associated dysfunction is observed in most, if not all, organs, and dysfunction in one organ can impair others via organ crosstalk. For instance, both CKD and HF are age-associated diseases that reciprocally promote disease. As they age, the kidneys undergo structural changes, such as decreases in nephron size and number as well as fibrosis, which together cause a decline in renal function (111). On top of the normal aging of the kidneys, the increasing prevalence of risk factors, such as hypertension and diabetes, increases the prevalence of CKD (112). The cardiovascular system also undergoes structural and histological changes with age, including vascular stiffening, increased left ventricular wall thickness, and fibrosis, leading to diastolic dysfunction (113). These age-associated changes may interact with each other, promoting dysfunction in both organs. Gut microbiota may also act as a component in age-related organ crosstalk. Intestinal dysbiosis was found in progeria patients and mouse models. Fecal microbiota transplantation from wild-type mice increased both the healthspan and lifespan in the progeria models (114), highlighting the contribution of the gut microbiome to aging.

**Figure 6:**
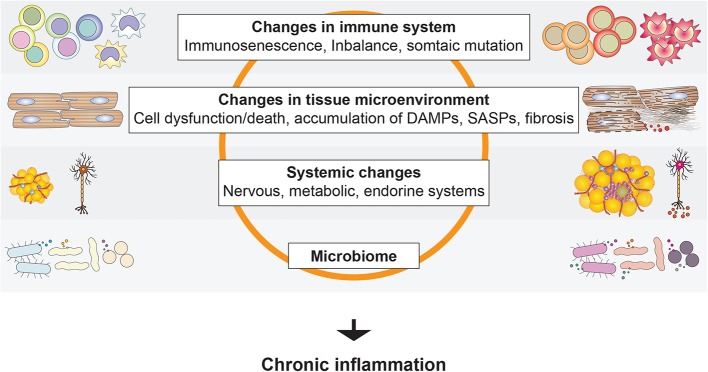
Activation of age-associated chronic inflammation (inflammaging). A variety of local and systemic factors have been suggested to promote inflammation in the elderly.

A feature of much age-associated organ dysfunction is tissue remodeling, such as fibrosis. Chronic inflammation plays a key role in the development of tissue remodeling and is a hallmark of age-associated diseases. Moreover, an elevated systemic inflammatory state is seen in aged subjects with, for example, elevated levels of proinflammatory cytokines, clotting factors, and acute phase reactants (115). Such chronic activation of inflammation associated with aging is termed “inflammaging” (59). The factors involved in the chronic activation of inflammation in the elderly include age-associated alteration in the immune and hematopoietic system, accumulation of DAMPs, senescence-associated secretory phenotype (SASP) factors produced by senescent cells, and somatic mutations (Figure 6). This is in addition to the factors that activate inflammation in younger adults, such as obesity.

Dysregulation of the sympathetic nervous system is another hallmark of aging and is also associated with obesity (116). As summarized in the previous sections, hyperactivation of sympathetic nerves directly and indirectly contributes to NCD progression. Given the tight connection between the immune and nervous systems, such hyperactivation of sympathetic nerves may contribute to inflammaging. Chronic inflammation therefore appears to be a key convergence point for age-associated changes in various systems and diseases.

### Cardioncology

An increasingly significant factor in multimorbidity is cancer. Cancer is a complex tissue containing both cancer cells and stromal components that include noncancerous stromal cells and extracellular matrix, and the complex interactions between the cancer cells and stromal components greatly influence tumor growth, progression, and metastasis (117). Indeed, two hallmarks of cancers are inflammation and angiogenesis, the main processes of which are played out within the stroma (118). As with organs, cancer can be affected by signals from distant organs and the body as a whole, and vice versa. For instance, diabetes and obesity are risks for many cancers (119). Conversely, cancer induces cachexia, which is not limited to skeletal muscle and involves multiple organs and systemic inflammation (120). Cancer is thus linked to various NCDs. One area rapidly gaining attention is the intersection of cardiology and oncology, cardioncology.

**Figure 7:**
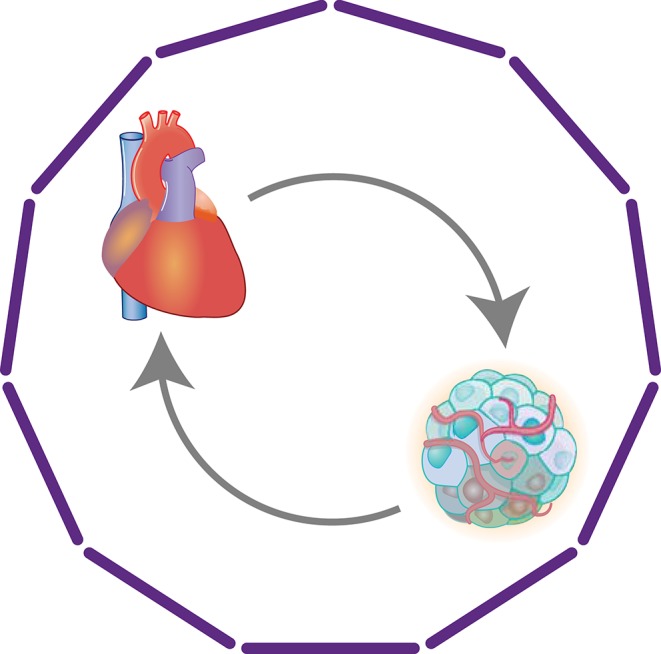
Heart-cancer crosstalk. Clinical studies have revealed reciprocal associations between HF and cancer. Although the cardiotoxicity of cancer treatments is a key factor in the increased morbidity and mortality from HF among cancer patients, multiple other mechanisms, including direct organ crosstalk, also appear to be involved.

The rapid advances in cancer treatments have resulted in increasing numbers of cancer survivors, who are known to be at higher risk for cardiovascular events (121, 122). The cardiotoxicity of cancer treatments is one of the main reasons for the increased morbidity and mortality due to CVD, particularly HF, among cancer patients (123). However, the link between cancer and HF appears not to be limited to the cardiotoxicity of the treatments. In fact, HF patients are at a higher risk for cancer (124–126), suggesting a reciprocal interaction between HF and cancer (Figure 7).

Cancer and HF share many risks, including diabetes, obesity, and hypertension, which may promote the comorbidity of both diseases (127). Chronic inflammation promoted by those risks is a likely connection. However, it is also possible that there is direct and indirect communication between the heart and cancer tissue. Meijers et al. showed that MI accelerated intestinal tumor growth in APC^min^ mice (128). They found that cardiac expression of *Serpina3* was increased in the mice and that SerpinA3 protein promoted proliferation of HT-29 colon cancer cells. Moreover, plasma SerpinA3 levels were also increased in HF patients. These results suggest that factors secreted from failing hearts promote cancer growth. Conversely, cancer may cause cardiac atrophy and remodeling (129, 130). Animal models, including the C26 colon adenocarcinoma mouse model, have also revealed associations between cancer and cardiac atrophy and remodeling (131, 132). Although the cancer-derived mediators of cardiac atrophy have not yet been identified, studies suggest that tumor-derived mediators, such as proinflammatory cytokines, promote muscle wasting (133, 134). These experimental data and clinical associations strongly suggest deeper connections in the organ crosstalk between cancer and the heart.

## Future Perspective

In this article, we have discussed several mechanisms that tightly link disease development in one organ with processes ongoing in multiple other organs and systems. The intricate connections among cells, organs, and organ systems highlight the needs to address interactions and networks involving multiple diseases and/or dysfunctions when analyzing even a single disease. Such studies are particularly important for understanding cardiometabolic disease, a key component of multimorbidity.

The strong connections among systems, such as the metabolic and immune systems, also suggest not only that these connections are attractive targets for novel therapeutic strategies, but also that there is a need to assess the therapeutic actions of drugs from the viewpoint of their effects on those networks of organs and/or systems. For instance, recent studies have demonstrated that a class of anti-diabetic drug, sodium glucose cotransporter 2 (SGLT2) inhibitors, has beneficial effects on heart failure and CKD in patients with or without type 2 diabetes (135–138). While the precise mechanisms remain elusive, multiple possible mechanisms for the beneficial effects of SGLT2 inhibitors have been proposed (139). In addition to their effects on hemodynamics, including reductions in preload and afterload, in part through natriuresis and osmotic diuresis (140), it has been suggested that these drugs exert anti-inflammatory effects on the heart and kidneys, and also have beneficial effects on myocardial energetics (141, 142). As such, the cardiac action of SGLT2 inhibitors could potentially involve the heart; arteries; kidneys; metabolic tissues, such as liver and adipose tissues; and immune cells, as well as the interactions among them mediated by cytokines, various metabolites, and sympathetic nerves.

Multimorbidity has become a serious medical, social, and economic burden and is increasing globally. This is likely driven in part by the graying of society, but also by other factors, including obesity, urbanization, and the growing burden of NCDs (6, 8). Rapid progress in the development of therapies also generates new clinical problems with multimorbidity, which are exemplified by cardioncology. Accordingly, elucidation of the mechanisms that connect multiple diseases and organs is becoming more and more important in this era of multimorbidity. However, most major research efforts, regardless of whether they are basic or clinical research, are still targeting a single disease or single tissue. Likewise, guidelines mostly focus on the management of a single disease. By comparison, very little attention is being devoted to coexisting multiple chronic conditions within one patient or model animal (6). Nonetheless, any CVD model is very likely to elicit responses in extra-target organs or the whole body that are also likely to crucially influence target organ disease development. Similarly, after acute MI, blood leukocytes and various inflammatory biomarkers are increased in patients, and the levels of some of them are known to be associated with adverse outcomes (143). This indicates that MI elicits systemic inflammatory responses, and these responses may modulate the disease's course. For those reasons, we will need to pay greater attention to the responses in the body and extra-target organs so as to better understand the intrinsic network of the systems involved in coping with the stress, but which may also propagate disease. Likewise, clinical studies are needed to elucidate and evaluate the roles of system interactions in disease progression and treatment.

The CANTOS trial was a landmark proof-of-concept trial in which inhibition of IL-1β signaling using canakinumab reduced the incidence of atherothrombotic events in patients with prior MI and a C-reactive protein (CRP) level ≥2 mg/L (144). Accordingly, that study as well as others (145, 146) support the notion that targeting inflammatory signals is an effective and feasible strategy against cardiometabolic disease. Interestingly, although it was not among the primary end-points, the incidence of lung cancer was significantly reduced in the canakinumab groups (147). The results suggest that targeting IL-1β inflammatory signaling may be beneficial for the prevention and treatment of lung cancer (148). As discussed in this article, chronic inflammation is a unifying mechanism in various NCDs and in the possible reciprocal interaction between the CVD and cancer. These findings may support the convergent position of chronic inflammation in CVD and cancer.

Various clinical studies, including many studies using statins, which may modulate immunometabolism, strongly suggest that targeting inflammation is a feasible and effective approach to the prevention and treatment of cardiometabolic diseases. However, several studies also provide caveats to such therapeutic strategies. For instance, TNF-α inhibition increased mortality and hospitalization rates in HF patients (149), while low-dose methotrexate failed to reduce cardiovascular events (150). These studies highlight the need for a better understanding of the timing and targets for interventions. As discussed, inflammation, even chronic inflammation, has essential protective and homeostatic functions. Consequently, interfering with inflammation might ultimately promote pathology. For example, even IL-1β has atheroprotective effects in mice (151), while GM-CSF appears to be cardioprotective in a mouse model of moderate-level left ventricular pressure overload (51). On the other hand, in a mouse model of MI, GM-CSF promotes inflammation resulting in adverse outcomes (152). Clearly, inflammation has many faces during the course of disease development and in different diseases. It is therefore essential that we better understand the many-faceted role of inflammation to identify the appropriate timing, disease state, and specific targets for therapeutic intervention on inflammation.

Here, we have provided an overview of the extensive connections between the immune and metabolic systems, the nervous and immune systems, and the nervous and metabolic systems. Recent studies have been sorting out the machinery that constitutes and executes these interactions, and it is likely that the coordinated interactions among these three systems are continuously operating in our bodies. The finding that the CNS response to insulin controls hepatic metabolism via vagus nerve activity that is relayed by Kupffer cells in the liver exemplifies such interactions (23). However, it largely remains unclear how the interactions among the immune, metabolic, and nervous systems both maintain homeostasis and contribute to CVD. Even when considering the interactions between two of these systems, the contributions to disease development remain insufficiently understood. For instance, it remains unclear whether the systemic metabolic disturbance caused by obesity and diabetes promotes chronic inflammation by modulating the cellular metabolism in immune cells. Obesity and diabetes alter the metabolic microenvironment, which includes nutrients, metabolites, and metabolic signals, and likely affects the cellular metabolism of immune cells within that environment. Metabolites generated from gut microbiota may also contribute to this altered metabolic microenvironment.

Technical advances surely stimulate research on organ–organ and organ–system crosstalk. For instance, new technologies such as optogenetics enable real-time activation and inhibition of specific nerves. In addition, at the tissue, single-cell, and molecular levels, barcoding technologies, mass microscopy, and tissue clearing could potentially enable fine mapping of cell–cell and cell–metabolite interactions.

In conclusion, research into organ–organ and organ–system crosstalk is increasingly important, given the growing clinical problem of multimorbidity. Studies of crosstalk mechanisms and their roles in cardiometabolic disease and cancer will identify novel therapeutic and diagnostic targets. It will also be important to promote clinical investigation into multimorbidity and the interactions between two or more diseases, which could accelerate basic and clinical study into reciprocal crosstalk.

## Author Contributions

YO and IM wrote the manuscript.

## Conflict of Interest

The authors declare that the research was conducted in the absence of any commercial or financial relationships that could be construed as a potential conflict of interest.
